# Insights on Antioxidant Assays for Biological Samples Based on the Reduction of Copper Complexes—The Importance of Analytical Conditions

**DOI:** 10.3390/ijms150711387

**Published:** 2014-06-25

**Authors:** Sara S. Marques, Luís M. Magalhães, Ildikó V. Tóth, Marcela A. Segundo

**Affiliations:** REQUIMTE, Departamento de Química, Faculdade de Farmácia, Universidade do Porto, Rua Jorge Viterbo Ferreira, 228, 4050-313 Porto, Portugal; E-Mails: micf09213@ff.up.pt (S.S.M.); luismagalhaes@ff.up.pt (L.M.M.); msegundo@ff.up.pt (M.A.S.)

**Keywords:** total antioxidant capacity, cupric ion reducing antioxidant capacity, serum samples, high-throughput microplate assay

## Abstract

Total antioxidant capacity assays are recognized as instrumental to establish antioxidant status of biological samples, however the varying experimental conditions result in conclusions that may not be transposable to other settings. After selection of the complexing agent, reagent addition order, buffer type and concentration, copper reducing assays were adapted to a high-throughput scheme and validated using model biological antioxidant compounds of ascorbic acid, Trolox (a soluble analogue of vitamin E), uric acid and glutathione. A critical comparison was made based on real samples including NIST-909c human serum certified sample, and five study samples. The validated method provided linear range up to 100 µM Trolox, (limit of detection 2.3 µM; limit of quantification 7.7 µM) with recovery results above 85% and precision <5%. The validated developed method with an increased sensitivity is a sound choice for assessment of TAC in serum samples.

## 1. Introduction

Antioxidants serve as a protection for the body against the harmful effects of free radical damage. Generally, the measurement of total antioxidant capacity (TAC) indicates the oxidant-buffering potential of a tissue or biofluid. However, the use of serum TAC as a biomarker of oxidative damage is frequently criticized [1,2]: regarding the non-concordant results between *in vitro* and *in vivo* assays [3]; the dependence of results obtained on the chemistry of TAC assay applied [4]; as oxidation probes, targeted molecules and measurement conditions differ across the assays used for plasma [5,6]. Practical methodological aspects (pH, monitoring wavelength, assay time) [7] also play an important role in the interpretation of the results of TAC assays.

One group of antioxidant capacity assays is based on the electron transfer (ET) mechanisms. The most widely used ones resort to Vis-spectrophotometry for monitoring the bleaching of colored radical compounds. Ferric reducing antioxidant power (FRAP) [8] and cupric ion reducing antioxidant capacity (CUPRAC) [9] are similar from a theoretical point of view, as both are based on the reduction of metal complexes.

In ET based methods, the reactivity is conditioned by the de-protonation and the ionization potential of functional groups. Therefore, ET reactions are pH dependent. Generally, ionization potential values decrease with increasing pH, which causes an increase in electron-donating capacity concomitant of de-protonation [10]. Hence, pH values have a major impact on the reducing capacity of antioxidants. At acidic conditions, the reducing capacity may be restrained or slowed down due to protonation on antioxidant compounds, whereas, in basic conditions, the dissociation of phenolic compounds with proton release will increase reducing capacity [11]. Regarding assays based on reduction of metal or on prevention of metal oxidation, redox active complexes of iron [8] and copper [9] are the most frequent choices as oxidant probes. 

For instance, the Ferric reducing antioxidant power (FRAP) assay is based on the reduction of the Fe(III) complex of 2,4,6-tripyridyl-s-triazin (TPTZ) by antioxidants and the intense color change induced by the appearance of the Fe(II)-TPTZ chelate is monitored at 593 nm. This assay has the advantage of using a spectrophotometric measurement at a wavelength far from the UV range where most samples (food, beverage or those with biological origin) have an intrinsic absorbance. 

The cupric reducing antioxidant capacity assays are based on the reduction of a Cu(II) complex the presence of antioxidants, in this case the color change is related to the reduced form of the complex which can be monitored at a visible wavelength. The advantages in using the cupric assay over the FRAP one include the working pH, which is closer to the physiological conditions (pH 7.0 in CUPRAC *vs.* the pH ~3 necessary in FRAP). In general the copper based assays target the thiol group containing antioxidant species. The chromogenic oxidizing reagent used for the assay is a copper(II) cation chelate acting as an outer-sphere electron-transfer agent [12], and the chromophore, formed by reduction of this reagent with antioxidants, is the chelate-copper(I) cation. Due to the lower redox potential of the reagent complex in comparison with the FRAP assay, oxidizable substrates such as reducing sugars and citric acid (not targeted in antioxidant assays) are not oxidized by the copper complexes [13]. Moreover, the low redox potential enhances redox cycling [14,15], thus, copper reduction can be used as a sensitive indicator of potential pro-oxidant activity of antioxidants [16,17].

Nevertheless, all copper reduction based assays applied to complex mixture of antioxidants have shown discrepant results related to the appropriate selection of reaction time [10]. This drawback can be handled in different ways. One way is stopping the reaction at a predefined reaction time, avoiding the need to wait until completeness of the reactions, which can be time consuming (>1 h) for slow acting antioxidants, or even impossible to achieve if recirculation (redox cycling) phenomena is considered. Therefore, Apak and his co-workers [9] opted to use a fixed reaction time (30 min) and Campos *et al.* [18] have chosen to stop the reaction by addition of a strong complexing agent (EDTA) after 3 min. Other possibility is to try to find a model compound (calibrator) with a kinetic behavior similar to that of the antioxidant pool present in samples [19,20]. This kinetic matching approach is based on the comparison of the oxidation behavior of standard compounds and was validated through the analysis of real complex sample matrices (red wine and samples of biological origin).

The monitoring wavelength used in these assays can also present some disadvantages when sample background absorption is not negligible. In spectrophotometric assays sample background absorption is usually reduced by sample dilution, as in the case of red wine samples where extensive dilutions are necessary [21]. In the case of considerable intrinsic absorbance of an antioxidant compound itself (like bilirubin) the contribution of this compound to the true total antioxidant capacity can be overestimated [22]. Dilution is also chosen as a strategy to reduce interference effects [23], as other complexing agents and metal ions present in the sample can hinder the real antioxidant capacity. Extensive dilution of the samples can bring another disadvantage to the assessment of total antioxidant capacity, as samples might contain powerful antioxidant species that even at very low concentration can contribute significantly to the total capacity of the sample, and the contributing effect of these compounds can be “lost in dilution”. 

Therefore our present work is focused on (i) developing of a high-throughput microplate assay with increased sensitivity to facilitate the assessment of antioxidant capacity at low antioxidant concentrations; (ii) evaluating the possibility of monitoring the presence of synergistic or autoxidative effects in the case of various antioxidant species present in samples of biological origin.

## 2. Results and Discussion

### 2.1. Complexing Agent Selection

**Figure 1:**
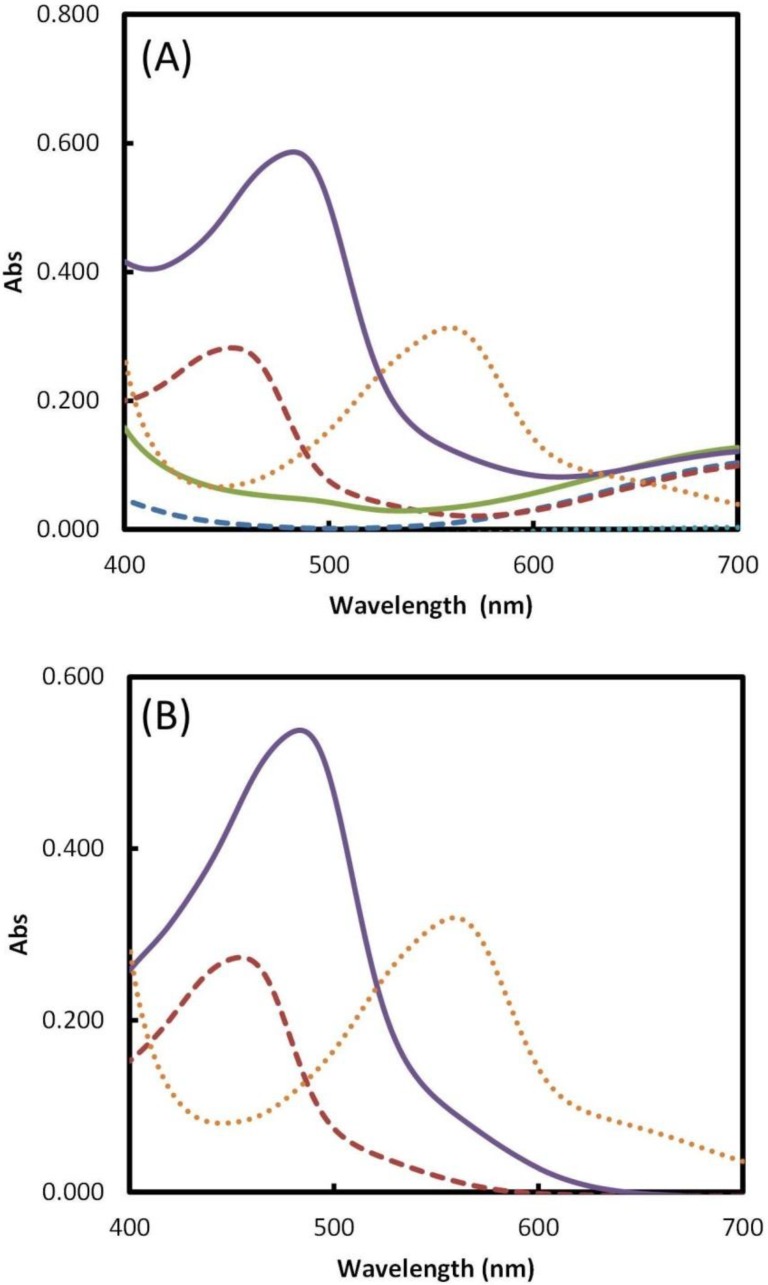
Absorption spectra for Cu(I) complexes and corresponding blank solutions (**A**); net absorption spectra (**B**); NC blank dashed blue line; NC in presence of ascorbic acid dashed red line; BCS blank solid green line; BCS in presence of ascorbic acid solid purple line; BCA blank dotted blue line; BCS in presence of ascorbic acid dotted orange line (reaction conditions given in Table S1 in presence of 50 µM ascorbic acid).

The absorbance spectrum in the visible region (400–700 nm) was traced for the three copper complexes in the presence of 50 µM ascorbic acid against the corresponding blank solution containing only the chelating agent, copper, and the buffer solutions (Figure 1A). The longest wavelength for maximum absorbance was found for the BCA complex (558 nm), however the highest absorption was verified for the BCS at 485 nm (Figure 1B). 

Phenanthroline derivate reagents (2,9-dimethyl-1,10-phenanthroline, NC, and 2,9-dimethyl-4,7-diphenyl-1,10-phenantrolinedisulfonic acid, BCS) are diamine chelators which form 2:1 complexes with Cu(II) at pH 7.0 (see structural formula within supplementary information Figure S1A,B). In the presence of a reducing agent, the strong complex stabilizes the copper in its reduced form of Cu(I) in a tetrahedral binding geometry [24], while the induced conformational changes of the complex result in the appearance of a strong absorption band in the 450 to 550 nm wavelength range. As the molecular mass of the chelating agent increases the absorbance band intensity also increases, as can be confirmed on Figure 1B for NC and for BCS.

Two molecules of bicinchoninic acid (Figure S1C) (2-(4-carboxyquinolin-2-yl)quinoline-4-carboxylic acid, BCA) will also chelate with copper ions and form a purple-colored product that strongly absorbs light at a wavelengths of 354 and 562 nm [25]. This reaction of BCA, whose mechanism is not fully understood yet, is widely used to assess protein concentration (Smith assay) [26].

All of the three complexes has high formational constants (logβ_NC_ = 19.5 [27]; logβ_BCS_ = 19.8 [28] and logβ_BCA_ = 14.7 [28]) showing high affinity towards Cu ions, assuring that the antioxidant assay will only evidence the reduction of the Cu complexes, and not Cu^2+^ in its free form. The formation of other metal complexes in the case of the BCS and NC assays could be ignored since excess concentration of the Cu(II) is used in the reaction mixture. The concentrations of Cu^2+^ (in the form of Cu aqua ions) present in the reaction mixture not complexed with NC or BCS is set at 2.5 mM. In the BCA assay, however, the concentration of Cu cannot be maintained in excess, since precipitation of the complex occurs [29]. To guarantee a complete complexation, without precipitation, the BCA must be added in stoichiometric ratio or in excess. Therefore, the potential complexation of other metal ions that might be present in the sample (for example Fe) cannot be ignored, and this situation limits the applicability of BCA. This effect was not noticed in the case of the other two complexants.

### 2.2. Reagents Addition Order

**Figure 2:**
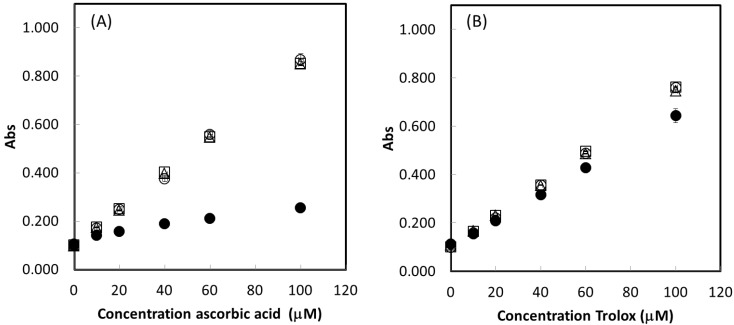
Response curves for ascorbic acid (**A**) and Trolox (**B**) antioxidants using different reagent addition orders, (a) (○) Cu^2+^, BCS, Buffer, AOX; (b) (□) Buffer, Cu^2+^, BCS; AOX; (c) (●) AOX, Buffer, Cu^2+^, BCS; (d) (Δ) AOX, Buffer, BCS, Cu^2+^.

In a first approach the time sequence for addition of the reaction components was tested. This study was carried out using BCS as a copper chelating reagent in the presence of Trolox and ascorbic acid as antioxidants, the other reaction conditions were as detailed in Table S1. Less than 2 min elapsed between the additions of each reaction component. The different addition orders tested were (a) Cu^2+^, BCS, Buffer, AOX; (b) Buffer, Cu^2+^, BCS; AOX; (c) AOX, Buffer, Cu^2+^, BCS; (d) AOX, Buffer, BCS, Cu^2+^. The step of buffer addition assured that the complexation and the reduction of Cu^2+^ occurred at the controlled pH of 7.0. The results obtained are represented in the form of calibration curves (0 to 100 µM) for ascorbic acid (Figure 2A) and for Trolox (Figure 2B).

One important detail concerning the analytical approach under these conditions is related to the order of addition of the reactants. Addition order effects can be significant, as can be concluded from Figure 2. The markedly different response of the addition order c (AOX, Buffer, Cu^2+^, BCS), can be traced back to the reduction of the free (non complexed) Cu^2+^ ions by the antioxidants before the complexation with BCS can take place. The different reduction potentials of 0.066 V for ascorbic acid *vs.* 0.192 V for Trolox in relation to the reduction potentials of the copper (*E*_Cu(II/I)_ = 0.17 V) and the copper-BCS complex (*E*_Cu(II/I)-BCS_ = 0.844 V) [30], is reflected in the different behavior find for the two antioxidant. The addition order c should be treated differently as it can lead to discrepancies in the results or to “apparent” interaction effects when various antioxidants are present in the sample solutions. Some of the literature data also indicate the occurrence of this phenomenon: in the presence of ascorbic acid in synthetic mixtures the found TE values were below the expected ones [31] and, in more recent articles, where this pre-reduction effect is exploited for the assessment of pro-oxidant behavior of ascorbic acid [16,17].

### 2.3. Trolox Equivalent Value

The relative response of the different antioxidants tested was established based on the Trolox equivalent values (TE) calculated according to Equation (1) in the experimental section. The obtained values are summarized in Table 1, and calibration curve parameters are exemplified in Table S2 in supplementary information.

**Table 1:** Trolox equivalent (TE) values obtained using different copper complexing reagents.

TE	Ascorbic Acid	Trolox	Uric Acid	Glutathione
TE_(BCS)_	1.15 ± 0.02	1.00 ± 0.01	1.90 ± 0.02	0.62 ± 0.02
TE_(NC)_	0.90 ± 0.03	1.00 ± 0.01	1.96 ± 0.02	0.54 ± 0.01
TE_(BCA)_	1.03 ± 0.03	1.00 ± 0.04	2.46 ± 0.09	0.57 ± 0.02

Results correspond to calibration curves performed in three working days for each complexing agents (5 standards in 0–100 µM antioxidant solutions each assessed in quadruplicate).

Confirming the additivity of the response given by the different antioxidant compounds present simultaneously in the samples is fundamental for a Total antioxidant capacity assay, as it will assure that the contribution of all the studied antioxidant compounds can be accounted for. Analytically, additivity is established if the sum of molar absortivities times the concentration of the antioxidants (TE_Calc_) equals to the experimentally determined absorbance value (Equation (2) in the experimental section).

This study is recognized as essential for the development of Total antioxidant capacity assays [9,31,32], however in the case of BCS complex a detailed study is missing from the literature [18,22]. In the present work this additivity was confirmed at two levels, (i) in binary mixtures of two antioxidants in sample solutions and (ii) in the simultaneous presence of the three biologically active antioxidants.

Covariance data is presented for the study of the binary mixtures of the antioxidants (Table S3), with the 95% confidence interval computed for each equation parameter; while in Table 2 the results obtained when three antioxidant compounds were simultaneously present can be found. 

**Table 2:** Calculated (TE expected) and determined (TE found) values for solutions containing three antioxidant compounds simultaneously using the BCS complex.

[AA] µM	[AU] µM	[GSH] µM	TE Expected	TE Found Average ± SD	RD% ^a^
10	10	10	0.037	0.038 ± 0.000	3.0
10	10	50	0.065	0.066 ± 0.000	1.7
10	50	10	0.111	0.112 ± 0.001	0.5
10	50	50	0.139	0.142 ± 0.001	1.8
50	50	10	0.157	0.158 ± 0.004	0.9
50	50	50	0.185	0.188 ± 0.001	1.9
50	10	10	0.082	0.085 ± 0.003	2.7
50	10	50	0.110	0.114 ± 0.001	2.9

^a^ RD%, Relative deviations (%) of the means: (TE found − TE expected) × 100/TE expected.

### 2.4. TE of Biological Antioxidants in Mixtures

The three complexing agents studied gave similar values for Trolox equivalent (Table 1), indicating the same order of “antioxidant power” for the studied compounds: uric acid > ascorbic acid ≈ Trolox > glutathione. Relative TE values based on the comparison to Trolox as a reference compound indicate 4, 2, and 1 electron transfer processes for uric acid, ascorbic acid and glutathione, respectively. Literature data in most cases indicate similar values; however the relative response of uric acid is not always coincident with the 4 electron transfer mechanism. This variation will reflect on the results for antioxidant capacity assays, and can justify the highly variable results obtained in biological samples [20,33,34] where uric acid constitutes an important antioxidant reserve. Based on the previous considerations the most sensitive reagent (BCS) was selected for the further studies.

No evident interaction of the antioxidants can be detected as the intercept and the slope values of the adjusted models were not statistically different from 0 and 1, respectively (Table S3). It is important to stress that in these assays care was taken to assure the shortest time possible (below 5 min) between the preparation of the antioxidant mixtures and the addition to the microplate. The same conclusions were found when the three antioxidant compounds were simultaneously present in different concentrations (Table 2), as relative deviations between the expected and found TE values were ≤3%. These results indicate the applicability of the method to total antioxidant capacity assays.

### 2.5. Buffer Solutions

**Figure 3:**
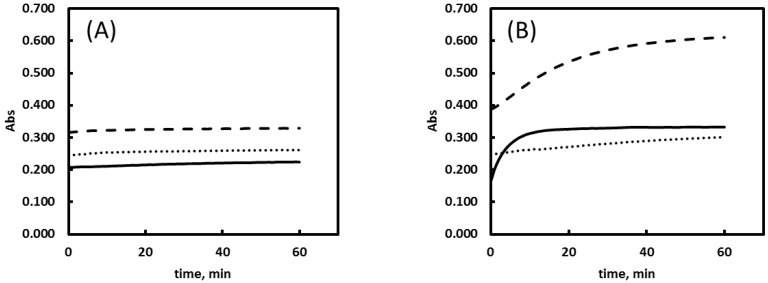
The influence of buffer solution composition on the absorbance readout for (**A**) Trolox and (**B**) uric acid at 40 µM. Dashed line is used for the ammonium acetate buffer, dotted line for PBS and solid line for the Tris-urea buffer. Other conditions are as detailed in Table S1.

The operating pH of Cu reducing TAC assays provides an advantage when biological samples are concerned compared to other ET assays. However the nature of the buffer solution can be variable, with reported utilization of PBS [18], Tris-glycine urea [34] and ammonium acetate [35]. In the present study, the response of the different antioxidants in these buffer solutions was evaluated and compared (Table 3). Figure 3 shows the different time course (kinetic response) of uric acid and Trolox in the three buffering systems.

In the case of biological samples the working buffer solution employed have important effects on the stability of the protein fraction and therefore on the obtained results. As the experimental conditions (namely the chosen chelating agent and the applied assay time) are very different in the literature-described applications, in the present work response of the different model compounds in the three buffer solution using the BCS reagent was evaluated during 1 h. The results indicate similar calibration curves for ascorbic acid, Trolox and glutathione, but uric acid shows a different behavior (Table 3).

**Table 3:** Comparison of Trolox equivalent values (TE) obtained for the antioxidant compounds, in different buffer solutions using BCS as a complexing agent.

Buffer	Ascorbic Acid	Trolox	Uric Acid	Glutathione
Ammonium acetate, pH 7.0	1.148 ± 0.024	1.000 ± 0.008	1.899 ± 0.013	0.624 ± 0.018
Tris-glycine urea, pH 7.0	0.813 ± 0.040	1.000 ± 0.050	0.983 ± 0.049	0.552 ± 0.028
PBS, pH 7.4	0.991 ± 0.044	1.000 ± 0.050	0.766 ± 0.038	0.668 ± 0.033

Results correspond to calibration curves performed using the three buffer solutions (5 standard antioxidant solutions in 0–100 µM concentrations range each assessed in quadruplicate).

Literature data is also divergent on the TE value of uric acid, Apak *et al.* initially reports TE_(NC)_ = 0.96 and 1.54, later Celic *et al.* [34] reported TE_(NC)_ = 1.050; Campos *et al.* [18] refers TE_(BCS)_ = 1.08 and in an earlier work of our group Ribeiro *et al.* [20] set the TE_(NC)_ at 1.74. The nature of the buffer solution seems to influence greatly the TE values. The oxidation process of uric acid can follow various pathways; Robinson *et al.* [36] postulate one main route of two electron transfer leading to the formation of allantonin, and a four electron transfer pathway leading to the formation of triuret. It is also proposed that triuret in the presence of oxidants will originate an amino carbonyl radical. Under the experimental conditions oxidants remain in excess in the reaction mixture at the time of detection. Therefore, this radical formation process (through the auto-oxidation of urate) is probably the cause for the kinetic curve of uric acid (Figure 3B) not reaching a constant value like in the other buffer solution. 

The Tris-glycine buffer with 8 M urea [37] contains also 4 mM citrate, therefore chelation of copper by the buffer components can also occur under the used experimental conditions [38]. Celic *et al.* [34] refers that the use of this buffer is necessary to avoid the precipitation of the protein fraction of the sample, and add that the presence of 8 M urea partly denaturates proteins and significantly lowers the reduction potential of disulfide/thiol couples in peptides facilitating thiol oxidizability. 

It is currently estimated that albumin contributes to 60%–70% of the antioxidant capacity of serum, either directly via free reducing thiol groups, or indirectly by protecting the antioxidants from oxidation [39]. In serum samples, quantifying the contribution of the protein fraction to the total antioxidant capacity can be a troublesome process. Frequently, the protein fraction is separated by precipitation, applying isoelectric precipitation with perchloric acid or trichloroacetic acid, or by salting out with addition of ammonium sulfate. The total antioxidant capacity can then be assessed on the protein free fraction [8,40]. An analytical method with ability to assess the TAC of serum samples without separation of the protein fraction has advantages providing (i) a simpler sample preparation procedure; (ii) the possibility of assessing protecting effects of protein fraction. The Tris-glycine buffer with 8 M urea has been used for TAC assay successfully [34], and showed linear response when different dilutions of the sample were analyzed. PBS and 1 M ammonium acetate buffers were only used in protein free samples. The ammonium acetate concentration applied in this latter buffer induces steric changes in the protein structure (salting out effects) therefore precipitation may be observed.

### 2.6. Sample Analysis and Validation

Analytical features of the developed method were calculated based on the calibration curves established at three working days (Table S3). Back calculated concentrations for linearity assessment are listed in Table 4.

**Table 4:** Back calculated concentrations of standards for ascorbic acid, uric acid, glutathione and Trolox.

Nominal Conc. (µM)	Ascorbic Acid (µM)	Uric Acid (µM)	Glutathione (µM)	Trolox (µM)
10	10.0 ± 0.7	10.0 ± 0.6	9.9 ± 0.5	10.5 ± 0.6
20	20.3 ± 0.9	20.0 ± 0.7	21.8 ± 0.7	20.2 ± 0.7
40	39.8 ± 2.0	39.6 ± 1.7	42.0 ± 0.9	40.6 ± 0.7
60	59.3 ± 2.6	60.3 ± 1.9	61.0 ± 1.3	61.0 ± 1.2
100	99.9 ± 2.8	100 ± 1.5	97.9 ± 1.9	100.9 ± 1.1

*n* = 12, corresponds to 3 working days and assays in quadruplicate.

The limit of detection (2.3 µM), was estimated from the standard error of the Trolox calibration curve [41], while the limit of quantification corresponds to 7.8 µM. The application range extends to 100 µM Trolox. Precision values obtained from calibration curves in 3 working days at five concentration levels and in quadruplicate assays for the four antioxidants studied are summarized in Table S4. Sample stability results assessed under different storage conditions are presented in Table S5. Recovery assay results with two level addition of uric acid in the different samples (*n* = 5) are summarized in Table 5.

**Table 5:** Recovery assay results for two level addition of uric acid in five serum samples.

Sample	Absorbance		TEAC (µM)		Recovery%
	Added Uric Acid Concentration		Trolox Equivalent (µM)		
	0 µM	25 µM	0 µM	44.1 µM	
S1	0.260 ± 0.002	0.524 ± 0.006	25.7 ± 0.2	66.7 ± 0.8	93.0
S2	0.271 ± 0.004	0.518 ± 0.006	27.4 ± 0.4	66.1 ± 0.7	87.9
S3	0.235 ± 0.005	0.493 ± 0.004	21.6 ± 0.4	62.2 ± 0.5	92.0
S4	0.265 ± 0.003	0.511 ± 0.005	26.4 ± 0.3	65.1 ± 0.6	87.7
S5	0.275 ± 0.004	0.520 ± 0.005	27.9 ± 0.4	66.5 ± 0.7	87.6

*n* = 8, corresponds to duplicate determinations in quadruplicate assays.

A serum sample of Standard Reference Material 909c of the National Institute of Standards and Technology (USA), containing 0.278 ± 0.006 mM of uric acid, was analyzed using the three buffer solutions to confirm their influence on results. Sample dilutions in the range of 50 to 200 times were prepared. However, samples diluted less than 100 times showed precipitation, resulting that the analytical signal was proportional to the turbidity of the solutions. Therefore, total antioxidant capacity of the sample (at 200 times dilution) was assessed based on the Trolox calibration curve established using the three buffer solutions, and the resulting TAC values for PBS, Tris-glycine urea, and ammonium acetate were 4.1 ± 0.5, 7.8 ± 0.6, and 1.75 ± 0.04 mM, (*n* = 3) respectively. Limitations imposed by the quantification limit did not allow higher than 200 times dilutions, and for sample inter-comparability in the validation procedure the sample dilution was fixed at 200 times. 

Stability of the standard solutions was not evaluated in this study as it is a consensus practice to use freshly prepared working solutions of the antioxidants. Sample stability results indicate that the matrix antioxidant compounds are stable during storage at −18 °C for at least 2 weeks. Four freeze-thaw cycles at ambient temperature during 1 h were also well supported. However, >10% decrease was observed for sample standing 24 h at room temperature (Table S5).

Recovery data indicates a good agreement (Table 5) as the recovery was >85% for the uric acid addition. Uric acid was used in this study since major contribution to the TAC of the samples is expected from this analyte. 

Regarding precision the % CV values in the study samples (1%–2.1%) were not different from the ones obtained for the standard solutions (1%–5.5%), indicating that the diluted sample matrix did not influence the method overall precision.

## 3. Experimental Section

### 3.1. Materials

All chemicals used were of analytical reagent grade and were purchased from Sigma Aldrich, MO, USA if not otherwise stated. Ultrapure water from Sartorius AriumPro system (resistivity > 18.2 MΩ·cm) was used in the preparation of all solutions.

For the microplate-reader method, 10 mM copper solution was prepared by dissolving CuCl_2_· 2H_2_O in water. Complexing agent solutions in water were prepared from neocuproine hydrochloride monohydrate (NC, CAS: 303136-82-5, Sigma 72090), bathocuproinedisulfonic acid disodium salt (BCS, CAS: 52698-84-7, Sigma B1125), and bicinchoninic acid disodium salt hydrate (BCA, CAS: 979-88-4, Sigma D8284) by dissolution in water in concentrations of 7.5, 7.5 and 6 mM, respectively. Ammonium acetate buffer (1.0 M) pH 7.0 was prepared by dissolving 3.85 g of ammonium acetate in 50.0 mL of water. In addition to the ammonium acetate buffer, phosphate and Tris-glycine-urea buffers were also prepared following the literature recommendations. For the phosphate buffer Dulbecco’s phosphate buffer saline (Sigma D1408) was used at 10 mM. The Tris-glycine-urea buffer contained 0.086 M tris(hydroxymethyl)aminomethane, 0.09 M glycine, 4 mM citrate and 8 M urea (Merck, Darmstadt, Germany) with the final pH adjusted to 7.0.

The stock solutions of the antioxidants were prepared daily. The stock solutions of 2.00 mM ascorbic acid (AA, CAS: 50-81-7, Fluka 95210) and 1.00 mM of reduced glutathione (GSH, CAS: 70-18-8, Sigma G6529) were prepared by dissolving the respective solids in water. Trolox (CAS: 53188-07-1, Fluka 56510) solution at 1.00 mM was prepared by dissolving 25.0 mg of the solid in 5 mL of ethanol followed by completing the volume to 100 mL with water. The stock solution of 1.00 mM uric acid (UA, CAS: 69-93-2, Sigma U2625) was prepared by suspending the corresponding solid in 20 mL water, the pH of the resulting solution was raised by drop-wise addition of 0.1 M NaOH solution until complete dissolution of the solid (pH 11–12), followed by the elimination of the excess of base by addition of 0.1 M HCl solution until reaching pH 7.0. Afterwards the total volume of the solution was completed to 100 mL with water. Working standard solutions of antioxidants in the range of 10 to 100 µM were prepared by adequate dilution in water. 

### 3.2. Instrumentation

Microplate reader was a BIOTEK, Synergy HT (Highland Park, VT, USA), with microplates of 96 wells (Orange Scientific Cat # 4430100, Braine-l’Alleud, Belgium), a glass pH electrode (Crison, 52-02, Barcelona, Spain) and potentiometer (Crison, pH meter GLP 22, Barcelona, Spain) were used, following the protocol detailed in Supplementary information (Table S1). Briefly, equal volumes (50 µL) of the complexing agent, copper solution and buffer solution were pipetted in this order and in quadruplicate into a micro well plate, followed by the addition of 100 µL the antioxidant solution (e.g., single standard, binary or tertiary mixtures or sample solution). Absorbance change was monitored during 1 h in 1 min intervals in the kinetic mode of the reader, for quantification purposes the absorbance values corresponding to the 1 h readings were used. 

### 3.3. Validation and Sample Analysis

The difficulty in validation of bioanalytical methods for multianalyte assays or for assays on parameters involving total concentration of various chemical identities is widely recognised [42]. Total antioxidant capacity assays fall outside the scope of guidelines published for validation of bioanalytical methods [43,44]. In the present work these guideline protocols were followed to assess the selectivity, accuracy, precision, recovery and sample stability of the developed method.

Selectivity in the present case cannot be assessed in the same manner as chromatographic separations as multiple analytes contribute to the total antioxidant capacity. It is, thus, important that the relative contribution of the different analytes is quantified. Therefore, analytical curves were established for the different analytes in study and their relative contribution was evaluated.

The relative response of the different antioxidants tested was established based on the Trolox equivalent values (TE). These values were calculated from the slope of the linear response curves (calibration curves) obtained for the antioxidants, therefore the value is the unity for Trolox (Equation (1)).

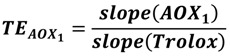
(1)


Linearity was evaluated using calibration curves obtained in 3 working days, with freshly prepared stock and working standard solutions. Precision and accuracy were evaluated through standard solutions and back calculated concentrations within the calibration range. Sample analysis precision was assessed from 2 replicate samples in quadruplicate assays (*n* = 8), after 200 times dilution.

Quantitative evaluation of matrix/mutual interference effect due to interactions of AO compounds was based on the comparison of the TE values obtained from the absorbance values of the mixed standard solutions (2 or 3 component) and the expected TEAC value based on the known concentrations of the antioxidants and their TE values (Equation (2)). The mixed standard solution preparation protocol is detailed within the supporting information (Table S6).

*TE_Calc._* = [*AOX*_1_] × *TE*_*AOX*_1__ + [*AOX*_2_] × *TE*_*AOX*_2__ + … + [*AOX_n_*] × *TE*_*AOX_n_*_ (2)

Lower and upper limits of quantification were estimated from the Trolox calibration curves established daily in three different working days [41].

Recovery assays were performed at two concentration levels using uric acid as model analyte. Absorbance values obtained before and after addition were compared to the ones expected after the addition of the known amount of the analyte, and taking into account the TE value of the uric acid. 

The evaluation of sample stability is a key topic in bioanalytical method validation [42]. To mimic the actual sample processing steps as recommended [43,45] aliquots of a sample were subjected to 3 repeated freeze-thaw cycles, long term (2 weeks) storage at −18 °C and a short term (24 h) room temperature storage. 

A serum sample of Standard Reference Material 909c of the National Institute of Standards and Technology (USA), containing 0.278 ± 0.006 mM uric acid, was analysed. Sample dilutions in the range of 50 to 200 times were prepared. Five study serum samples (from healthy volunteers) were also used for validation purposes.

## 4. Conclusions

Total antioxidant capacity methods based on spectrophotometric detection are important for the analysis of biological samples, and experimental conditions of Cu reducing assays are clearly advantageous over other methods based on the same principle. However, some important conditions have to be met so that the results can be conclusive and intercomparable.

- An additive response should be provided for the targeted group of antioxidants in the applied concentration range, because as long as the additivity is verified, the nature of the used Cu complexing agent does not seem to influence the results;
- Additive response is provided as long as the oxidant complex is maintained in molar excess considering the electron transfer processes;
- Selection of the buffering system affects more the TE values that the complexing agent used, and this is related to the electron transfer mechanisms of the antioxidants;
- The selection of buffer solution is also important to avoid measurement artefacts due to protein precipitation in biological samples.

There is a need for further mechanistic investigations focused on antioxidant interactions in relation to health effects in humans. The copper reduction based spectrophotometric methods are sensitive markers to detect changes of total antioxidant capacity, which may not be detectable through the measurement of single “specific” antioxidants. Therefore we believe that the copper reduction based total antioxidant capacity assays under strictly controlled experimental conditions is an important tool in assessing serum TAC.

## Supplementary Files

Supplementary File 1

## Supplementary Information

Supplementary data associated to this manuscript include: Table S1, Summarized experimental conditions for the studied copper reducing antioxidant assays; Figure S1, Structure of applied complexing agents; Table S2, Calibration curves obtained using the BCS complex for the studied antioxidant compounds; Table S3, Equation parameters, covariance data for assays containing binary mixtures of antioxidants; Table S4, Intra- and Inter assay precision for ascorbic acid, uric acid, glutathione and Trolox; Table S5, Stability of sample assessed under different storage conditions; Table S6, Preparation of binary mixtures of antioxidants.
