# Supplementary material for: Insights on Antioxidant Assays for Biological Samples Based on the Reduction of Copper Complexes—The Importance of Analytical Conditions
Source: Int J Mol Sci. 2014 Jun 25;15(7):11387–402. doi: 10.3390/ijms150711387 (PMC4139788; doi:10.3390/ijms150711387)
Supplement: Supplementary File 1 [file ijms-15-11387-s001.pdf]

Supplementary Information

Table S1. Summarized experimental conditions for the studied copper reducing antioxidant assays.

Reagent	Volume	Neocuproine (NC) [a]	Bathocuproine (BCS) [a]	Biciquionic Acid (BCA) [a]
Cu^{2+}	50 µL	10 mM	10 mM	2.0 mM
Reagent	50 µL	7.5 mM	7.5 mM	6.0 mM
Buffer	50 µL	1.0 M	1.0 M	1.0 M
AOX	100 µL		0–0.1 mM	
Wavelength		450 nm	485 nm	558 nm
Assay time			1 h	
Assay pH			7.0	

[a] Concentration values prior to addition to micro plate wells.

Figure S1. Structure of applied complexing agents, (**A**) Neocuproine (2,9-dimethyl-1,10-phenanthroline) NC; (**B**) Bathocuproine disulfonic acid (2,9-dimethyl-4,7-diphenyl-1,10-phenantrolinedisulfonic acid) BCS; (**C**) Bicinchoninic acid (2-(4-carboxyquinolin-2-yl)quinoline-4-carboxylic acid) BCA. The chemical structure information for these molecules is available in the PubChem Substance and Compound database through the unique chemical structure identifier NC-CID 65237; BCS-CID 170300 and BCA-CID 71068.

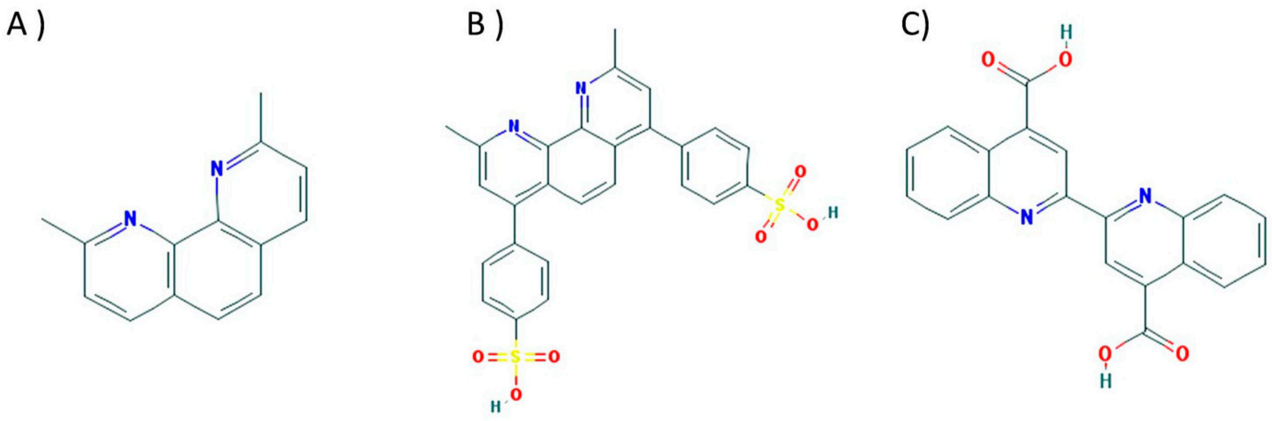

Table S2. Calibration curves obtained using the BCS complex for the studied antioxidant compounds.

Antioxidant	Intercept ± Standard Error [a]	Slope ± Standard Error [a], μM^{-1}	R^2
Trolox	0.103 ± 0.002	$(6.50 \pm 0.04) \times 10^{-5}$	0.9998
Ascorbic acid	0.101 ± 0.002	$(7.44 \pm 0.03) \times 10^{-5}$	0.9999
Uric acid	0.103 ± 0.002	$(12.24 \pm 0.04) \times 10^{-5}$	0.9999
Glutathione	0.118 ± 0.006	$(4.1 \pm 0.1) \times 10^{-5}$	0.9960

The estimated overall regression parameters correspond to the results obtained in separate working days (5 working concentrations, $n = 4$). [a] Estimate of the linear regression parameters (for 5 freshly prepared standard solutions in the range of 0 to 100 µM analyzed in quadruplicate).

Table S3. Equation parameters, covariance data (TEdet. = a + b × TEcalc.) for assays containing binary mixtures of antioxidants.

Composition [a]	a [b]	b [b]	*R*
Trolox/ascorbic acid	0.002 ± 0.001	0.96 ± 0.04	0.9983
Trolox/uric acid	−0.001 ± 0.001	1.00 ± 0.03	0.9991
Trolox/glutathione	0.002 ± 0.002	0.99 ± 0.04	0.9981
ascorbic acid/uric acid	−0.004 ± 0.002	1.02 ± 0.02	0.9996
ascorbic acid/glutathione	0.001 ± 0.002	1.10 ± 0.04	0.9990
uric acid/glutathione	0.002 ± 0.002	1.04 ± 0.03	0.9994

[a] n = 12 different solutions (Table S6) containing the two antioxidants in different ratios were analysed in quadruplicate; [b] value ± confidence interval (α = 0.05).

Table S4. Intra-, Inter assay precision for ascorbic acid, uric acid, glutathione and Trolox.

Conc. (μM)	Ascorbic Acid		Uric Acid		Glutathione		Trolox	
	WD%	BD%	WD%	BD%	WD%	BD%	WD%	BD%
10	1.2	7.0	0.6	5.7	1.7	4.0	1.1	5.3
20	0.5	4.2	0.7	3.5	1.2	2.9	1.1	3.5
40	1.1	4.7	0.4	4.3	1.1	1.8	0.9	1.5
60	0.8	4.5	0.7	3.0	1.2	1.8	0.8	1.7
100	1.3	2.4	0.4	1.5	0.9	1.7	0.6	0.9

WD% intra (within day) precision, BD% inter (between day) precision, n = 4, d = 3.

Table S5. Stability of sample assessed under different storage conditions.

Sample	Initial	2 Weeks at −18 °C	3 Freeze/Thaw Cycles	24 h Room Temperature
Average (μM TE)	28.9	27.7	27.6	25.6
SD	0.5	0.4	0.4	0.4
Variation	-	−4.1%	−4.5%	−11.4%

Table S6. Preparation of binary mixtures of antioxidants.

Assay No.	$[AOX_1]$/μM	$[AOX_2]$/μM	Ratio of $[AOX_1]/[AOX_2]$
1	10	10	1
2	20	10	2
3	30	10	3
4	40	10	4
5	50	10	5
6	10	20	0.5
7	10	30	0.3
8	10	40	0.25
9	10	50	0.2
10	20	20	1
11	30	30	1
12	40	40	1

Solutions were prepared by dilution of 0.2 mM antioxidant solutions in a final volume of 1.0 mL.
